# Animal Models of Hepatitis B Virus Infection–Success, Challenges, and Future Directions

**DOI:** 10.3390/v13050777

**Published:** 2021-04-28

**Authors:** Yongzhen Liu, Stephanie Maya, Alexander Ploss

**Affiliations:** 110 Lewis Thomas Laboratory, Department of Molecular Biology, Princeton University, Washington Road, Princeton, New Jersey, NJ 11 08544-101, USA; yongzhen@princeton.edu (Y.L.); smaya@princeton.edu (S.M.)

**Keywords:** hepatitis B virus, hepatitis B, animal model, species tropism, humanized mice

## Abstract

Chronic hepatitis B virus (HBV) infection affects more than 250 million people worldwide, which greatly increases the risk for terminal liver diseases, such as liver cirrhosis and hepatocellular carcinoma (HCC). Even though current approved antiviral therapies, including pegylated type I interferon (IFN) and nucleos(t)ide analogs, can effectively suppress viremia, HBV infection is rarely cured. Since HBV exhibits a narrow species tropism and robustly infects only humans and higher primates, progress in HBV research and preclinical testing of antiviral drugs has been hampered by the scarcity of suitable animal models. Fortunately, a series of surrogate animal models have been developed for the study of HBV. An increased understanding of the barriers towards interspecies transmission has aided in the development of human chimeric mice and has greatly paved the way for HBV research in vivo, and for evaluating potential therapies of chronic hepatitis B. In this review, we summarize the currently available animal models for research of HBV and HBV-related hepadnaviruses, and we discuss challenges and future directions for improvement.

## 1. Introduction

Hepatitis B virus (HBV) infections pose a major public health burden throughout the world, with a 3.5% global prevalence in the general population and over 250 million chronically infected individuals worldwide [1,2]. The clinical outcome of HBV infection in humans varies with age; while acute hepatitis and spontaneous viral clearance is common in adults, chronic infections develop in about 90% of perinatally-infected infants or children [3]. Persistent and chronic HBV infections can result in severe liver disease, including liver fibrosis, cirrhosis, and increased risk of developing hepatocellular carcinoma (HCC), which is responsible for about 887,000 deaths annually [4].

**Figure 1:**
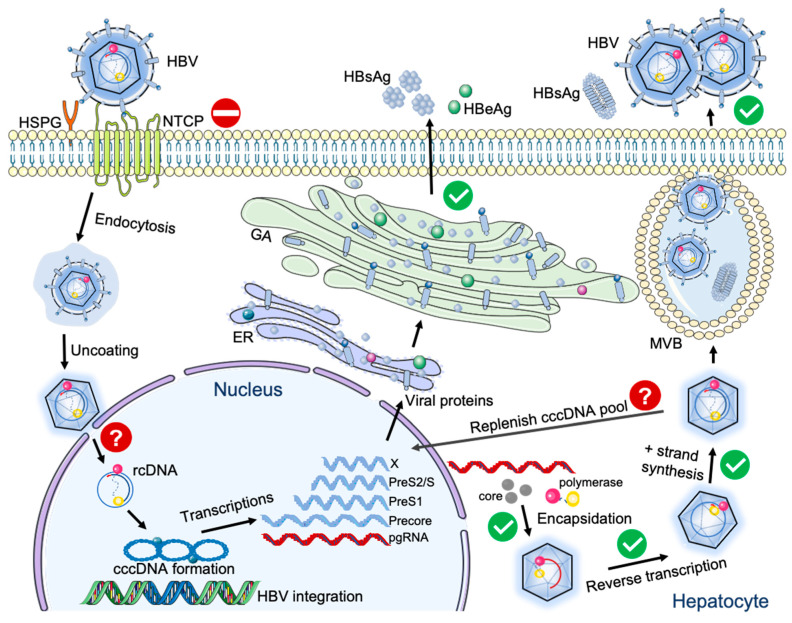
HBV life cycle and barriers to HBV species tropism. HBV is highly species-specific and hepatotropic. NTCP acts as an entry barrier to infection in monkeys and mice (denoted with red dash mark), while the nuclear pore may serve as a barrier against nuclear import in mice (denoted with question mark). Green checkmarks denote life cycle steps supported in monkeys and mice. There is still an incomplete understanding of how rcDNA is released into the nucleus and cccDNA replenishment in the nucleus of murine hepatocytes, denoted with a red question mark. HBsAg, HBV surface antigen; HBeAg, HBV e antigen; rcDNA, relaxed circular DNA; NTCP, sodium taurocholate co-transporting polypeptide; HSPG, heparan sulfate proteoglycan; GA, Golgi apparatus; ER, endoplasmic reticulum; MVB, multivesicular bodies.

HBV belongs to the *Hepadnaviridae* family and contains a partially double-stranded relaxed circular DNA (rcDNA) of approximately 3.2 kilobases in length that is generated through a unique reverse transcription process. The HBV genome is very compact; the rcDNA contained in the nucleocapsid is composed of four overlapping open reading frames (ORFs), including pre-core/core (preC/C), polymerase (P), pre-surface/surface (preS/S), and X, which are later transcribed into 3.5, 2.4, 2.1, and 0.7 kb polyadenylated HBV RNAs, respectively, with the same 3′ end (Figure 1) [5,6]. A distinctive characteristic of HBV is that it forms a stable mini-chromosome, denoted covalently closed circular DNA (cccDNA), in the nuclei of infected hepatocytes. cccDNA acts as the episomal viral genome and the template for all HBV RNA transcription, whose persistence accounts for the failure of viral clearance, a lack of a functional cure, and, particularly, for relapses after treatment cessation [7].

HBV is thought to be a non-cytopathic virus, and its pathogenesis is generally believed to be largely due to host immune response mediated liver injury. Although, viral components, such as the HBV e antigen (HBeAg), HBx, and the three HBV surface antigens (HBsAg), inhibit the host immune response, which contributes to HBV chronic infection [8]. While an efficient prophylactic vaccine for HBV is available, hundreds of millions of people worldwide are chronically infected, and, since the refractory nature of HBV intracellular replication intermediate cccDNA, combination therapies utilizing interferon in combination with nucleos(t)ide analogs rarely achieve elimination of the infection or a functional cure, i.e., the permanent inactivation of the virus in infected cells. Moreover, there is evidence of virological relapse in patients who have discontinued antiviral treatment [9,10], and hepatitis flares during and following cessation of antiviral therapies [11,12,13], as well as resistance to the lamivudine [14,15]. This suggests that chronic hepatitis B (CHB) patients may need life-long treatment due to the refractory nature of HBV, and more efficient HBV antiviral therapies may need to be deployed in combination with current treatments to promote immune restoration, such as check point inhibitors or adoptively transferred chimeric antigen receptor T cells, in the future [3,7,16].

Several HBV cell culture-based systems have been developed, including but not limited to HepG2T14 [17], HepG2.2.15 [18], Q7 HBV-21 [19], HepG2-4A5 [20], and HepAD38 [21]. These have been used to produce the virus, studying the regulation of viral replication and potential HBV inhibitor screening in vitro. However, dissecting mechanistically HBV pathogenesis, HBV-induced immune responses and testing of novel antiviral therapeutic regimens has depended and will continue to depend on in vivo models. Altogether, HBV in vivo infection systems are of particular importance for the field to wholly understand the mechanisms of chronic HBV infection, study the interplay between virus and host, and investigate new therapeutic strategies. This knowledge will aid the development of efficient antiviral therapies that deplete HBV infection and bolster immune regulation.

## 2. HBV Host Tropism

Hepadnaviruses have been discovered in several mammalian species (orthohepadnaviruses), including but not limited to: woodchucks (WHBV), woolly monkeys (WMHBV), ground squirrels (GSHBV), and tent bats (TBHBV), as well as in birds (avihepadnaviruses), such as ducks (DHBV), herons (HHBV), and Ross’ geese (RGHBV) [22,23,24]. Distinct viral lineages from amphibians, such as snakes (eSHBV) and frogs (TFHBV), were also found, revealing a complex evolutionary history for hepadnaviruses [25]. In 2016 and 2017, Nackednaviruses, which are non-enveloped HBV-related viruses, were found in teleost fishes [26,27]. Although the Nackednaviruses further widened the viral family host range, viruses in teleost fishes notably do not exhibit a marked liver tropism.

As a hepatotropic virus in the hepadnavirus family, HBV displays a narrow host range, robustly infecting only hepatocytes in humans and higher primates, such as chimpanzees [28,29,30]. Clinically, HBV inoculation in adult chimpanzees also leads to typical cases of acute, self-limited HBV infections and recover through immune-mediated viral clearance [31]. Tree shrews (*Tupaia belangeri*) also support HBV infection despite its evolutionarily distance from humans and great apes [32,33]. However, tree shrews are difficult to procure as they only live in the tropical forests of Southeast Asia and experimental HBV infections in these animals require specific conditions, such as immunosuppression prior to HBV inoculation, which hinder their usage as a common animal model for HBV infection [34,35]. Evidence for additional natural interspecies transmission of human HBV has been reported in cynomolgus monkeys (Table 1) [36]. Moreover, human HBV can be experimentally transmitted into gibbons [37,38]. It is worth noting that gorillas harbor HBV-like variants that can recombine with HBV from chimpanzees [39], indicating a possibility for recombination events and/or potential cross-species transmission with human HBV; however, further research is necessary to clarify this. More analysis on human HBV recombination events and testing of more animals, specifically non-human primates, are warranted in order to fully understand the potential for natural human HBV interspecies transmission.

The limited host and hepatocellular tropism of HBV hampers its mechanistic study and new drug preclinical assessments in vivo [28,29,30]. The experimental use of chimpanzees is heavily scrutinized, and there is a lack of small animal models that reproduce human-like HBV infections. This creates a major bottleneck for elucidating potential mechanisms of common and serious HBV disease and developing effective therapies.

Although an HBV mouse model might be ideal, various complications in this system arise. The first obstacle for HBV infection in murine hepatocytes was overcome by the discovery of the HBV and HDV receptor, sodium taurocholate co-transporting polypeptide (NTCP) [40]. Expression of human NTCP (hNTCP) in murine, rat, and dog hepatocytes enables HBV entry, but the virus is then blocked in one or more steps in the viral life cycle for reasons still unknown. Substitution of residues 85–87 of murine NTCP (mNTCP) with those of human NTCP is sufficient to facilitate entry into the cell [41]. On the other hand, hNTCP expression in cynomolgus macaque, rhesus macaque, and pig hepatocytes enables productive HBV infection, indicating that the barriers experienced in mice are not an issue in macaques [29]. Notably, amino acid residue 158 in hNTCP determines species specificity towards HBV infection in Old World Monkeys (OWM); substitution of this residue to that of cynomolgus macaque NTCP impedes HBV infection [42].

Once inside the murine hepatocyte, HBV is restricted in one or more steps along its life cycle, which are yet to be fully characterized (Figure 1). Transfection of HBV genomes into murine hepatoma cells resulted in production and egress of infectious HBV [43], indicating that the steps following cccDNA generation, namely viral protein synthesis, reverse transcription of pregenomic RNA (pgRNA), and virion assembly/secretion, are not blocked in mice. Heterokaryons formed from murine hepatoma cells expressing hNTCP and human hepatoma cells were susceptible to HBV infection [43], further indicating that there are possible missing factors critical for the HBV life cycle in murine hepatocytes. hNTCP-expressing transgenic mice have been successfully developed, and were shown to support HBV uptake in vivo using labelled virions [44](Figure 1). Furthermore, HBV cccDNA can be successfully formed in an immortalized murine hepatocyte cell line AML12HBV10, which harbors an inducible HBV genome and overexpresses human transforming growth factor (TGF)-α [45]. In this particular cellular environment, HBV nucleocapsids (NCs) were destabilized, facilitating the release of rcDNA and ultimately promoting cccDNA formation. When compared with hepatocyte nuclear factor (HNF1)α-expressing HBV transgenic mice, HBV cccDNA was detectable in HNF1 α-null HBV transgenic mice [46]. However, it is still unknown what leads to the destabilization of mature NCs in AML12HBV10 cells or hepatocyte physiological state changes in HNF1 α-null HBV transgenic mice, which may account for HBV cccDNA formation. Therefore, a natural HBV infection in mice is unattainable since HBV is unable to complete its life cycle in murine hepatocytes. For this reason, either surrogate models (discussed in Section 3), primate models (Section 4), and humanized mice (Section 6) provide viable approaches for attaining a reliable in vivo platform of acute and chronic HBV infections. Further research investigating the restriction factor(s) against HBV infection in murine hepatocytes is imperative in order to obtain a mouse model that can be directly infected with HBV and result in acute and/or chronic infections.

## 3. Surrogate Models Based on HBV-Related Hepadnaviruses

HBV is the prototypic member of the *Hepadnaviridae* family, which contains closely-related hepatotropic viruses in other species (Table 2). By employing the endogenous polymerase reaction of HBV and the autoradiography approach developed by Summers and colleagues, new hepadnaviruses were discovered in woodchucks (WHBV), domestic ducks (DHBV), and Beechey ground squirrels (GSHBV), respectively [47,48,49]. Another hepadnavirus with a host intermediate between humans and rodents was isolated from the woolly monkey (a New World primate), and it was designated as WMHBV [50]. At that time, these findings fulfilled the need for animal models that were necessary to elucidate the mechanisms of HBV replication and made a great contribution for HBV research.

### 3.1. Woodchuck Hepatitis B Virus

The discovery of woodchuck hepatitis virus (WHBV), a hepatitis B-like virus that infected Eastern woodchucks (*Marmota monax*) in the Philadelphia Zoo [48], opened up a new avenue for studying host responses towards hepadnaviruses in vivo. WHBV is similar to HBV not only in terms of its virological characteristics, including genome size, organization, nucleotide sequence (60~70% similarity), and protein expression, but also in the host innate and adaptive immune responses that occur upon viral infection [87]. Now, two types of woodchucks with known liver and spleen transcriptomes have been developed and applied as animal models for HBV-related research since they are highly susceptible to WHBV. One is the American woodchuck (*Marmota monax*), with high rates of chronic hepatitis and HCC following WHBV infection [59,88], while another is the Chinese woodchuck (*Marmota himalayana*), for which breeding colonies have been established [89]. Woodchucks infected with WHBV follow a similar natural history of infection, pathogenesis, and liver disease progression from chronic hepatitis to HCC compared with HBV infection in humans. Consequently, the woodchuck model has been widely used for preclinical evaluation of antiviral drugs and HBV-related HCC research. As preclinical models, woodchucks have been tested for antiviral drugs, such as nucleos(t)ide analogs, including but not limited to lamivudine [90], entecavir [60], and tenofovir [91]. Furthermore, experimental infection of newborn woodchucks usually leads to chronic infection, while adult woodchucks generally develop acute hepatitis, indicating a similar immune response against viral infection to humans. This finding improved the application of this animal model for prophylactic vaccines and screening of immune therapeutic strategies, such as programmed death ligand-1 (PD-L1) antagonists and toll-like receptor (TLR)7 agonists, against HBV infection [59,88,92,93].

New strategies for targeting WHBV are further being investigated. For example, it has been shown that the administration of cationic liposomes containing JVRS-100 (complexes of cationic liposomes and non-coding DNA) into WHBV-infected woodchucks deterred new tumor formation in the liver [91]. Recently, another group injected HCC-bearing woodchucks with nanoparticles as a potential approach to delivering antiviral drugs to the cancerous liver, finding that the nanoparticles localized in the liver and spleen and accumulated in macrophages [94]. Nevertheless, it should be noted that there are still various limitations in conducting research with the woodchuck model. They are difficult to handle and there are few existing reagents available to investigate the immune response against viral infection. In addition, the carcinogenesis between HBV and WHBV may be different, since WHBV DNA frequently integrates into the Myc proto-oncogene resulting in nearly all neonatally-infected woodchucks developing HCC, whereas HBV shows preference for integrating into TERT, MLL4, and CTNNB1 genes [95]. Thus, both virus and host-related differences pose considerable challenges and need to be taken into account for evaluating the efficacy of drug and vaccine candidates directed against HBV.

### 3.2. Duck Hepatitis B Virus 

In 1980, DHBV was detected in serum of domestic ducks and has become an instrumental model for understanding the hepadnavirus life cycle [49]. With duck hepatocyte cultures and ducklings available, this model played a pivotal role in elucidating the mechanisms of viral replication, including viral capsid assembly, initiation of reverse transcription, and finally rcDNA formation [92,93,96,97,98]. Specifically, by using this model, the steps for cccDNA formation were also probed, thus aiding our understanding of the establishment of cccDNA pools and its mechanism of replenishment within the nucleus [61,99,100]. Moreover, duck models with persistent DHBV infection were also widely used to evaluate antiretroviral drugs, nucleocapsid assembly inhibitors, and combined therapeutic strategies [62,101,102]. Nevertheless, DHBV and duck models still differ greatly from HBV and humans in the following aspects: DHBV is only 40% homologous related to human HBV [22], DHBV utilizes carboxypeptidase D as the entry receptor [103], and ducks may experience different effects from drug toxicity [104,105]. Consequently, drug screening and mechanistic research from this system may need further verification due to the viral and host differences.

### 3.3. Woolly Monkey Hepatitis B Virus

Another HBV-like virus, the woolly monkey hepatitis B virus (WMHBV) infects its natural host, the woolly monkey (*Lagothrix lagotricha*) [50]. This finding is exciting since woolly monkeys represent a host intermediate between humans and rodents that can be investigated for antiviral therapies for hepadnavirus infection. However, although the woolly monkey provides an additional arm for studying HBV-like viruses in animal models, woolly monkeys are endangered [106]; thus, little research has been conducted on the animals themselves. WMHBV, however, has been successfully utilized in various studies in spider monkeys (*Ateles geoffroyi*) and squirrel monkeys (*Saimiri sciureus*). A WMHBV infectious clone was designed for investigation in spider monkey models [104], resulting in moderate viremia (10^4^–10^5^ GE/mL) eight weeks post inoculation followed by immune-mediated clearance. However, spider monkeys are also endangered [107] and, consequently, are not commonly available for HBV-related research. Interestingly, immunodeficient liver injury mice engrafted with primary hepatocytes from squirrel monkeys were selectively susceptible to WMHBV as opposed to HBV [64]. Nonetheless, this non-human primate model may prove promising; squirrel monkeys infected with WMHBV exhibited acute infections and, in some instances, chronic infections with the help of adeno-associated virus (AAV)-mediated delivery of WMHBV [64]. 

### 3.4. Hepadnavirus Infections in Tupaias

Tupaias, also known as treeshrews, are small, non-primate-like mammals that are genetically more closely related to primates than to rodents [33]. Tupaias are experimentally susceptible to HBV and HCV, leading to them becoming a non-primate animal model that has been well developed in recent decades [34,108]. Similar to other species, tree shrews have a greater propensity of progressing to chronicity when infected as neonates and exhibit similar liver histopathological changes as those in HBV-infected humans [58]. Since Tupaia primary hepatocytes are fairly widely available, they have become widely used for HBV and WMHBV infections [40,109]. However, to date, there are a number of caveats that limit the applicability of Tupaias as an animal model, including their genetic heterogeneity as an outbred species (which may be useful to represent real-world situations but are not ideal for large-scale animal experiments), the overall low viral titers in vivo, and the scarcity of research tools and materials for this species. Nonetheless, Tupaia hepatocytes still prove useful as demonstrated by their transplantation into chimeric mice (Section 6), resulting in efficient HBV infection [110].

## 4. HBV-Susceptible Primate Models 

### 4.1. Chimpanzees

The chimpanzee is the only known immunocompetent non-human primate model that is fully susceptible to human HBV (Table 1) and can quite precisely mimic the pathogenesis and disease progression caused by HBV in humans. It has been shown that even one genome equivalent (GE) of HBV DNA was sufficient to successfully infect chimpanzees [54]. After inoculation with HBV derived from chronic hepatitis B (CHB) patients, chimpanzees can develop acute and chronic HBV infection with immune response profiles and inflammation much like those of HBV-infected patients [57].

In the early stages of HBV research, the chimpanzee animal model played an important role in evaluating the safety and efficacy of HBV vaccine candidates. Chimpanzee studies were used to test the efficacy of the first-generation (plasma-derived) HBV vaccine and the later-developed vaccine containing HBsAg from yeast [51,57]. In recent years, efficacy of the modified recombinant vaccines, vaccines against antiviral drug-resistant HBV mutants, and some therapeutic vaccination studies were also tested by incorporating the chimpanzee model [52,53,55].

As an immunocompetent animal model that can experience similar liver inflammation and cellular immune responses to HBV-infected patients, chimpanzees seem irreplaceable in HBV-related immune research. Studies in chimpanzees revealed that non-cytopathic antiviral mechanisms mediated by inflammatory cytokines may contribute to viral clearance during acute viral hepatitis [31]. More recently, research in CHB chimpanzee models revealed that immune modulation could enhance antiviral immunity and suppress HBV replication (e.g., via TLR-7 activation by the agonist GS-9620) [56]. Although chimpanzees were historically the most important animal model for HBV research, the constraint in availability, high-associated costs, and considerable ethical concerns have restricted their use as an experimental model.

### 4.2. Smaller Non-Human Primates

Continuous efforts have been made to establish HBV infection animal models in smaller non-human primates (NHPs). One naturally occurring transmissible HBV strain with 99% identity to an HBV genotype D (ayw) strain was discovered in 2013 in a cynomolgus macaque population on the island of Mauritius (mcHBV), which raised some hopes for developing a small, old-world monkey model [36]. However, this finding is still controversial since no productive infection occurred, even though the cynomolgus macaques were challenged with high doses of mcHBV [111]. 

After hNTCP was identified as the functional receptor for HBV, researchers found that NTCP was the key host factor limiting HBV infection in cynomolgus and rhesus macaques [29]. While hNTCP differs by only five amino acids within the HBV binding region between humans and macaques, one of these amino acid differences (G158R) leads to the failure of HBV binding to macaque NTCP, and conversely, adaptive substitution at this residue (converting the monkey NTCP to the human sequence (R158G)), was sufficient to confer HBV susceptibility [42]. Utilizing the widely used and efficient genome editing tool, CRISPR/Cas, an R158G gain-of-function mutation could be introduced into macaques NTCP so as to produce a new HBV primate animal model. 

A physiologically relevant rhesus macaque model was established that can support HBV infection following viral vector-mediated in vivo expression of human NTCP [111]. In this study, either helper-dependent adenovirus (HDAd) or adeno-associated virus (AAV) vectors expressing hNTCP were injected into rhesus macaques followed by HBV challenge, resulting in sustained HBV viremia for at least 6 weeks. This model can form cccDNA in macaque hepatocytes and successfully induce humoral and anti-viral cellular immunity, such as HBc antibodies and HBV-specific T cell responses, respectively. However, since they experience low hNTCP expression, this rhesus macaque HBV infection model is not efficient since only 0.5–1.0% of hepatocytes are HBcAg-positive, while the commonly assessed serological markers HBsAg were not detected [111]. These data are highly encouraging to pursue so that other smaller non-human primate animal models can be developed that exhibit higher HBV infection efficiency.

## 5. Non-Infection Murine Models

### 5.1. HBV Transgenic Mouse Model

HBV transgenic mouse models have been generated expressing either HBV proteins (such as HBsAg [66], HBeAg [70], or HBx [69], to investigate their pathogenic roles), or whole HBV genomes, which can produce infectious HBV virions in murine hepatocytes [44,71,72,112]. These models made particularly vital contributions in identifying the oncogenic functions of HBsAg and HBx by inducing inflammation, altering host gene expression, and eventually leading to the development of HCC [67,69]. Since the HBV transgenic mice were integrated with HBV genomes and thus could produce HBV virions in the peripheral blood, these transgenic models have also been employed to test the efficacy of antiviral drugs, such as lamivudine and entecavir, and to evaluate antiviral effects of small interfering RNA (siRNA) therapeutics that target HBV transcripts [73,113]. HBV transgenic mice are immunotolerant to HBV, since the HBV genome is integrated into the mouse genome and thus propagates HBV, similarly to infants who experience mother-to-child vertical transmission [114]. However, as an immune competent animal model, HBV transgenic mice have also been utilized for HBV-related immune research, including investigating the antiviral effect of the IFN-α/β inducer polyinosinic-polycytidylic acid (poly(I-C)) [115] and TLR 7/8 agonist CL097 [72], suggesting that the HBV transgenic mouse model can be widely used for many in vivo HBV preclinical antiviral evaluations. Additionally, adoptive transfer of immune cells from naïve mice into transgenic mice can instigate immune responses towards HBV [116,117,118]. One study crossed HBV transgenic mice strains harboring null alleles for recombinase-activating gene 1 (Rag-1) or the T cell receptor (TCR C-α) to eliminate most or all tolerized immune elements [117]. These mice were then used as recipients for adoptive transfer of splenocytes from non-immunized syngeneic donors, which led to acute or chronic hepatitis in Rag-1^−/−^ or TCR C-α^−/−^ HBV transgenic mice. Mechanistic work-up showed that liver inflammation in this model was primarily caused by nonclassical NKT cells (CD1d restricted but nonreactive to α-Galactosylceramide) [117]. Splenocytes were also adoptively transferred into young and adult Rag1^−/−^ HBVEnv mice (HBVEnvRag mice) or Rag1^−/−^ HBVRpl mice (HBVRplRag mice) [118]. The young mice developed strikingly weak HBV-dependent inflammatory responses compared with adult mouse recipients. Mechanistically, these studies found that higher HBV-dependent IL-21 production, lymphoid organization facilitated by hepatic macrophages, and immune priming in adult mouse livers may play pivotal roles in determining age-dependent immune response potency upon HBV infection [118,119,120].

### 5.2. Viral Vector-Mediated HBV Transduction 

To investigate HBV-specific immune responses that cannot be examined in traditional HBV transgenic mice, viral vector-mediated transduction of HBV DNA into immunocompetent mice has been demonstrated and can sustain viremia for several months before immunological clearance of the virus (Table 2). By using this strategy, AdV or AAV vectors containing HBV genomes are delivered to mouse livers in order to establish self-limiting acute HBV infection or to mimic a situation resembling chronic infection for mechanistic research of immune-mediated HBV clearance [121,122,123,124]. Generally speaking, in these viral vector-mediated HBV transduction mouse models, HBV cccDNA is not formed robustly and conceivably not through canonical rcDNA repair steps. HBV cccDNA has been detected in the liver of AAV-HBV transduced C57BL/6 mice, even though HBV cccDNA could alternatively originate from AAV-HBV episome recombination [125]. Interestingly, studies utilizing the AdV or AAV method of HBV expression in mice found that immunotolerance was developed, indicated by the failure of the murine immune system to produce anti-HBsAg antibodies [124,126]. In addition, the T cell landscape in the liver was altered by the induction of regulatory T cells (Tregs). Thus, the viral vector-mediated HBV transduction approach lends itself for the continued use in immunotolerance studies for HBV. Moreover, delivery of AAV-HBV vectors into mice yielded histopathological manifestations consistent with liver fibrosis [127]. It is important to note that the construction of the vectors might alter the manifestations of liver disease and this matter should be further probed. 

### 5.3. Delivery of HBV Genomes through Hydrodynamic Tail Vein Injections (HDI) into Mice

HDI is an effective technique for inducing transient expression of genetic material in the murine liver through intravenous injections of plasmids encoding the HBV genome in large (ca. 8% of body weight) volumes of saline over a short period of time (5–7 s) (Table 2). By injecting HBV replication-competent materials, for instance, the 1.2×HBV, 1.3×HBV genome, or HBVcircle (cccDNA-like plasmid), HBV viremia can be sustained for more than one week or even longer depending on the HBV strain [128,129,130]. One such study even reported infection for up to six months through hydrodynamic delivery of an AAV-HBV vector [131]. Since the viral genome is not integrated into the immune-competent mouse genome, this system also allows for investigation of immune control and assessment of specific CD8+ T cell subpopulations with potent antiviral activity under HBV infection conditions [132]. Aiming to achieve persistent infection, HDI of recombinant cccDNA plasmids into mice notably resulted in liver fibrosis [133]. In HDI-based mouse model studies, CRISPR/Cas9 genome editing has also been employed to destroy HBV-expressing vectors and reduce viral replication [130]. Combining HBV transgenic mouse models, HDI, and CRISPR/Cas9 genome editing technology, these mice can be used for mechanistic research of HBV-related HCC [134]. The comparation of HBV genome expression systems in mice with different methodologies was summarized in Table 3.

## 6. Humanized Xenotransplantation Models for the Study of HBV 

### 6.1. Human Liver Chimeric Mice 

**Figure 2:**
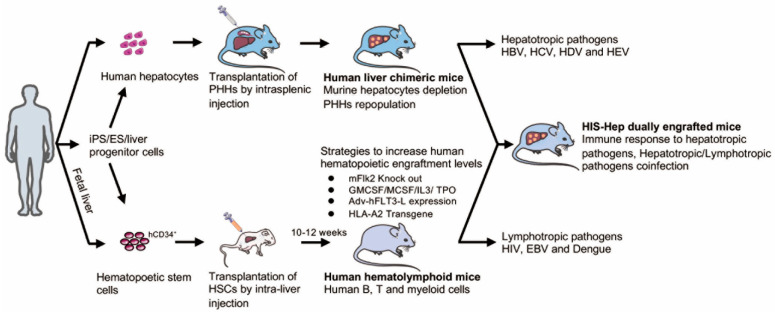
Humanized xenotransplantation models for the study of HBV. To produce human liver chimeric mice, hepatocytes are isolated from human subjects and transplanted to appropriate immunodeficient mice by intrasplenic injection. Due to the liver injury in the recipient, human hepatocytes can proliferate in the mouse livers yielding animals with high hepatic chimerism, which can be used for the study of HBV or other hepatotropic pathogens. For the human hematolymphoid mice, CD34+ human hematopoetic stem cells (HSC) are injected into immunocompromised xenorecipient mice. After 10 to 12 weeks, many cellular lineages of the human immune system (HIS) including but not limited to B, T cells, and some myeloid lineage cells can be reconstituted in the human hematolymphoid mice, which can be used for lymphotropic pathogen infection study. To improve human hematopoietic level, a variety of approaches have been taken as detailed in the text. To obtain donor matched hepatocytes or HSCs fetal tissue or iPS or ES cell derived populations can be used but those engraft usually considerably less efficiently Dual engraftment of human hepatocytes and HSCs yields HIS-Hep dual engraft mice, which can be used to study the immune response to HBV or other hepatotropic pathogens or to study hepatotropic/lymphotropic pathogen co-infection.

Arguably one of the best models for studying HBV persistence are humanized xenotransplantation models (Figure 2) in which the murine liver is re-populated with human hepatocytes [74,76,81,136]. To facilitate engraftment, suitable xenorecipients must be immunodeficient to prevent graft rejection, and must also suffer from an endogenous liver injury to promote expansion of the transplanted hepatocytes. Robust engraftment of human hepatocytes has been shown in a number of immunodeficient liver injury models and will be detailed below, including: Alb-uPA [137,138], *fah*^−/−^ [78,79], HSV-TK [82], AFC8 [139], MUP-uPA [140], and NSG-PIZ [141] mice. Of these, the former three are the most commonly used and will be discussed in the following paragraphs.

In mice harboring an albumin promoter with a urokinase-type plasminogen activator (Alb-uPA mice), overexpression of the uPA transgene is hepatotoxic, thus stimulating proliferation of transplanted human hepatocytes, which are readily rejected when crossed to the recombinase activating gene 2 (Rag2^−/−^) background, in which functional B and T cells do not develop [74]. Human liver chimeric Alb-uPA Rag2^−/−^ mice have been shown to be susceptible to HBV infection [74]. However, since human hepatic chimerism was overall rather low and not sustained for very long periods of time due to the residual anti-graft response on the Rag2^−/−^ background, efforts were made to further immunocompromise Alb-uPA mice by crossing them with different backgrounds (e.g., the severe combined immunodeficient (SCID) or the loss of the uPA transgene due to chromosomal rearrangement in mice carrying the uPA transgene heterozygously [142], as well as death by hemorrhaging [143] due to transgenic uPA expression). To circumvent these issues, a number of different approaches have been taken, including the expression of uPA under the major urinary protein (MUP) promoter [140] and injection of SCID mice with embryonic stem cells containing a cDNA-uPA transgene in order to attain optimal uPA activity. HBV inoculation of these mice proved fruitful, with sustained viremia 70 days post infection [75]. Efficacy of this particular model was further probed in a large-scale effort using 386 uPA/SCID mice and 493 cDNA-uPA/SCID mice that were injected with HBV. This study found higher human hepatocyte repopulation, continuous viremia, and increased health in cDNA-uPA/SCID mice as opposed to those lacking the cDNA transgene [136]. 

However, the frailty of the uPA model impedes the throughput at which human liver chimeric mice can be generated. To overcome this caveat, alternative xenorecipient strains have been generated in which murine hepatocytes can be more selectively ablated. One such model are fumaryl acetoacetate hydrolase deficient *(fah*^−/−^*)* mice initially crossed to the Rag2^−/−^ interleukin 2 receptor γ chain null (*IL2Rγc^null^*) background, which enabled more controllable induction of liver injury in a highly immunocompromised strain [76]. Fah deficiency results in a build-up of fumarylacetoacetate (FAA), an intermediate in tyrosine catabolism that is cytotoxic at high concentrations. Prior to transplantation, mice can be maintained on 2-(2-nitro-4-trifluoro-methylbenzoyl)1,3-cyclohexedione (NTBC) that inhibits an enzyme upstream of fah, thereby preventing the build-up of FAA to hepatotoxic concentrations. Consequently, liver injury can be induced by simply withdrawing NTBC from the drinking water, creating a hepatic environment that is suitable for human hepatocyte expansion. [77]. Furthermore, this triple mutant mouse model also lacks functional B and T cells and natural killer (NK) cells as a consequence of the Rag2 and *IL2Rγc* deficiencies, respectively [76]. Human liver chimeric *Fah^−/−^ Rag2^−/−^ IL2Rγc^null^* (FRG) mice, when successfully infected, supported HBV viremia for at least 7 weeks post inoculation [78]. 

Taking the idea of this model one step further, *fah^−/−^* mice were crossed to *NOD* *Rag1^−/−^ IL2Rγc^null^* (NRG) mice to produce FNRG mice. Because of their more severe immunodeficiency—phagocytic cells on the NOD background exhibit decreased anti-human cell activity—, human hepatocyte engraftment is more robust in FNRG mice than in FRG mice [79]. Human liver chimeric FNRG mice sustain high levels of persistent HBV infection. Notably, treatment of chronically infected FNRG mice with the HBV reverse transcriptase inhibitor, entecavir, efficiently suppressed HBV viremia. Upon treatment cessation, HBV infection rebounded, reminiscent of patterns observed in chronic HBV carriers. [144]. Pretreatment of FNRG mice with retrorsine, a pyrrolizidine alkaloid that suppresses hepatocyte proliferation, resulted in further enhanced human hepatic chimerism [80,145]. 

Finally, TK-NOG mice were generated by transgenically expressing herpes simplex virus type 1 thymidine kinase (HSVtk) in NOG mice, which are devoid of functional B cells, T cells, and NK cells [81]. This model overcomes the added risk of liver injury seen in the previously described models since it does not use liver-damaging drug treatments after human hepatocyte transplantation. Instead, ganciclovir (GCV) is used to selectively deplete cells that express the HSVtk transgene, as HSKtv converts GCV into a toxic metabolite, thus allowing for HSVtk-free donor cell engraftment [81]. 

To determine if the TK-NOG mouse model was amenable for HBV infection studies, one study inoculated TK-NOG mice and uPA-SCID mice with HBV from human samples. HBV viremia was successfully established in both types of human liver chimeric mice, which responded similarly well to entecavir or interferon treatment [82]. Interestingly, the particular HBV genotype may dictate the course of infection in TK-NOG mice [146]. Mice hydrodynamically injected with plasmids containing HBV genotype A efficiently established persistent infection while those containing genotype C could not [146]. Undoubtedly, these finding will have been expanded to other genotype C strains to ascertain the generality of these observations [146]. It was further shown that knockdown of NTCP in the donor human hepatocytes in TK-NOG mice abrogated HBV viremia [147], which is consistent with prior studies using HBsAg-derived peptides to block infection in human liver chimeric uPA mice. 

### 6.2. Dually Engrafted Mice

A considerable caveat of the human liver chimeric models is their highly immunodeficient background. Thus, in order to enable analysis of human immune responses to HBV, virally induced immunopathogenesis, and testing of immunomodulators for the treatment of chronic hepatitis B, protocols are being continuously refined to co-engraft mice with human hepatocytes and components of a human immune system (HIS). Dual humanization of both the liver and immune system has been achieved by co-transplantation of human hematopoietic stem cells (HSCs) and either adult [84,148,149,150] or fetal [83,139,151] hepatocytes (Figure 2). 

Historically, it has been rather challenging to achieve high levels of human hepatic chimerism with anything but adult hepatocytes. Fetal livers offer one of the few opportunities to obtain donor-matched hepatic cells and HSCs. However, fetal hepatoblasts do not respond to the same extent to growth stimuli in the injured murine liver, presumably due to their immature phenotype. To overcome this challenge, FNRG xenorecipients were pretreated with human oncostatin M (OSM), a pleiotropic cytokine belonging to the interleukin 6 group of cytokines, which plays a critical role in hepatocyte maturation. Indeed, OSM-treated mice became more robustly engrafted with human hepatic cells following human fetal hepatoblast injection. Co-injection donor-matched hepatoblasts and HSC yielded not only B and T cells, but notably also increased frequencies of monocytes and NK cells; however, while HBV infection stimulated NK cell proliferation, it did not affect the frequency of T cells circulating in the mice [83]. Therefore, further improvements on this foundation are warranted in future studies. HIS-HUHEP mice, which are engrafted with components of a human immune system (HIS) and human hepatocytes (HUHEP), were designed based on the *BALB/c Rag2*^−/−^ *IL2R*γ^NULL^ *Sirpa*^NOD^Alb-uPA^tg/tg^ (BRGS-uPA) background [84]. HBV infection resulted in sustained viremia over the course of 20 weeks and, curiously, human albumin levels rose in singly-engrafted HUHEP mice, whereas those in HIS-HUHEP mice did not [150]. Histological analysis showed T cells surrounding HBV-infected cells and macrophages dispersed throughout the liver parenchyma. Entecavir treatment significantly suppressed HBV viremia and liver inflammation.

HSCs and liver progenitor cells have also been introduced into livers of *HLA-A2 NOD-SCID-IL2Rγ^NULL^* (A2/NSG) mice in which liver injury was inflicted via the administration of an anti-Fas antibody [85]. This convenient humanized method yielded dually engrafted mice supporting HBV infection for more than three months in the presence of a human immune system. Although this A2/NSG/Fas-hu HSC/Hep model shows limited evidence for antigen-specific T cell responses in the liver, it will serve as a foundation for continued work in chronic HBV infection studies. 

### 6.3. Recent Progress and Improvements of Humanized Mouse Models in Other Fields

Humanized mice clearly serve as an invaluable tool for a better understanding of viral infections in humans and, importantly, how the immune system can affect these pathogens. Improvements on these models in other fields, such as in hepatitis C virus (HCV), have bolstered the advancement of humanized mouse models. 

Other xenotransplantation models in addition to the ones mentioned above have been created for studying other hepatitis viruses. Expression of a caspase 8 FK506 binding domain (FKBP) fusion protein on the *BALB/c Rag2^−/−^ IL2R*γ^NULL^ mice enabled the selective ablation of murine hepatocytes. Consequently, transfer of human liver progenitor cells and HSCs resulted in the dual engraftment of human liver and immune system. AFC8-hu HSC/Hep mice supported HCV infection and showed evidence of liver fibrosis [139]. Mice with immune system and human liver, denoted as HIL mice, were also developed utilizing human fetal liver progenitor cells as to overcome the need for treatment for transplantation [152]. HCV infection of HIL mice resulted in liver fibrosis and carcinoma within 4 months [153], a remarkably short period of time when compared to the slow disease progression of chronic hepatitis C in humans. Furthermore, the addition of HCV-specific immune system components into these models, such as KIR3DS1+ NK cells [154], has pushed the field further in characterizing immune responses to HCV. 

### 6.4. Future Directions for Humanized HBV Mouse Models 

The currently available HBV-related in vivo infection animal models are summarized in Table 2. Considering that HBV is a non-cytopathic virus and hepatitis B is largely a host-specific immune-mediated liver disease, the dually engrafted mouse model with simultaneous engraftment of both hepatocytes and HSCs is an ideal small animal model for HBV-related research and may be more acutely required in the future [155]. However, the dual chimeric mice, particularly those with efficient reconstitution of the human immune system, is hampered by several logistical and technical difficulties. For instance, the limitations inherent in HIS mice originate from the different growth factors and cytokines required for human hematopoietic and immune system development, and the presence of mouse MHC versus human HLA molecules. As a result, with the remaining mouse innate immunity in the humanized mouse model, species-specificity of homing molecules may hinder the appropriate trafficking of engrafted human immune cells. 

Several advances in the field have started to address some of these challenges (Figure 2). Previous work showed that human IL-15 and Flt-3/Flk-2 ligand expression in the recipient mice by hydrodynamic tail-vein injection or adenovirus-mediated plasmid delivery could elevate the level of NK cells and other myeloid cell populations by using NSG or NRGF mice, respectively [156,157,158]. In addition, physiologically relevant levels of human interleukin-7 (hIL-7) in humanized mice could promote homeostatic proliferation of both adoptively transferred and endogenously generated T cells [159]. Replacement of mouse genes with the corresponding human genes encoding non-cross-reactive cytokine, including thrombopoietin, interleukin 3, macrophage colony stimulating factor (CSF), and granulocyte macrophage CSF (GMCSF), aided in human HSC maintenance and foster myeloid cell lineage development [160]. Genetic ablation of MHC class I- and MHC class II genes in xenorecipient strains has helped to reduce graft-versus-host reactivity following engraftment of components of a human immune system [161,162]. Additionally, the development of functional adaptive immune responses is limited by the lack of human leukocyte antigen (HLA) gene expression. Expressing a human MHC class I allele has multiple benefits as it allows for more faithful development of CD8+ T cells in the thymus, enables recognition of (viral) antigens in peripheral tissues by human CD8+ T cells, and facilitates tracking of antigen-specific CD8+ T cells with MHC multimers, as previously shown for Epstein–Barr virus (EBV) and dengue virus infections [163,164,165]. Likewise, it has previously been suggested that expression of HLA-DR4, a human MHC class II molecule, partially improves the development of functional human T and B cells [166,167]. Adaptive immune response to adenovirus infections in humanized HLA-A*0201 and HLA-DRB*01 doubly transgenic mice, significantly improved the clearance of viral antigens from the liver [168]. Additionally, introduction of human thymic and/or lymphoid tissue can aid in T cell development and priming of adaptive immune responses, respectively. 

Humanized mouse models also lend themselves for modelling clinically relevant co-infections [169,170,171,172,173]. At least 15–20 million people are co-infected with HBV and HDV, 8 million with HBV and HCV, and 2 million with HBV and HIV, and even triple and quadruple infections of the viruses listed have been previously reported [174,175]. These complex viral interactions and how these co-infections affect HBV-induced liver disease remain poorly understood. Co-infection with HBV and HIV may accelerate the progression of liver disease and increased liver-associated mortality when compared with HBV mono-infection, while the immunodeficiency caused by HIV enhances the likelihood of HBV and HCV persistence [176]. The susceptibility of singly engrafted HIS or HUHEP mice to lymphotropic (e.g., HIV, EBV, Kaposi’s sarcoma herpesvirus (KSHV), or human T-lymphotropic virus (HTLV)) and hepatotropic (e.g., HBV, HCV, HDV) infections suggests that the novel dually humanized mouse model may be a robust platform to investigate the viral pathogenesis of mono-infections, as well as co-infections, and to evaluate novel therapeutic strategies and/or vaccine candidates.

Humanized mouse models may also show promising value for other co-morbidities with HBV infection. Hepatic steatosis cases have been increasing at an alarming rate [177] and this can lead to inflammation and fibrosis, resulting in the more serious condition known as non-alcoholic steatohepatitis (NASH), which in turn increases the risk of HCC. A recent study reported that human liver chimeric TIRF (Transgene-free *IL2rγ*^−/−^/*Rag2*^−/−^/*Fah*^−/−^) mice developed signatures of human clinical non-alcoholic fatty liver disease (NAFLD) in the engrafted human hepatocytes after they fed the mice with a Western-type diet [178]. Since both NAFLD and CHB is commonly observed particularly accompanied by the growing prevalence of NAFLD [179], humanized mouse models may be useful to imitate NAFLD and HBV infection simultaneously for potential mechanistic studies and drug development.

## 7. Conclusions

The surrogate, non-human primate, and mouse models described here have provided the field with a multitude of answers concerning the HBV life cycle, pathogeneses, and host immune responses towards the virus. With the reduction of chimpanzee usage and difficulties in working with woodchuck, duck, tree shrew, and woolly monkey models, a mouse model for HBV is necessary for future HBV research. We still lack a complete understanding of how the immune response attacks the virus in acute infections and how HBV can circumvent the immune system in chronic infections. The incorporation of murine non-infection models and humanized xenotransplantation models have greatly aided our understanding of which immune components are involved in acute and chronic HBV infections. With continued efforts to reproduce an immunological environment similar to that of humans, humanized mouse models can be accurately developed for future HBV studies in drug therapeutics and for developing a functional cure.

## Figures and Tables

**Table 1:** Overview of orthohepadnaviruses found in primates. Various primates contain species-specific HBV variants that can potentially undergo interspecies transmission. A major block for natural HBV infections in certain non-human primates is the expression of particular amino acid residues in the NTCP receptor. Potential recombination events have been evidenced in humans, chimpanzees, gibbons, and gorillas.

	Natural HBV Variant:	Permissive to	Barrier for Natural HBV Infection:	Potential Recombination Events with:
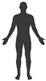 Human	HBV	HBV	None	chHBV; gibHBV
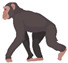 Chimpanzee	chHBV	HBV chHBV	None	HBV; gorilla-specific HBV variants
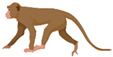 Cynomolgus monkey	Possibly mcHBV	HBV (in hNTCP-expressing hepatocytes)	158R residue of NTCP receptor	N/A
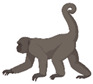 Woolly monkey	WMHBV	WMHBV	NTCP receptor	N/A
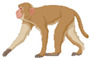 Rhesus macaque	N/A	HBV (in hNTCP-expressing hepatocytes)	158R residue of NTCP receptor	N/A
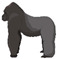 Gorilla	Gorilla-specific HBV	Gorilla-specific HBV; Possibly chHBV	N/A	chHBV
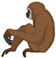 Gibbon	gibHBV	gibHBV	N/A	HBV
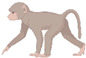 Squirrel monkey	N/A	WMHBV	Unknown (contains 158G of NTCP receptor, like human)	N/A
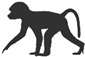 Spider monkey	N/A	WMHBV	Unknown	N/A

chHBV, chimpanzee HBV; mcHBV, cynomolgous HBV; WMHBV, woolly monkey HBV; gibHBV, gibbon HBV; hNTCP, human sodium taurocholate co-transporting polypeptide.

**Table 2:** Animal models developed for HBV study.

Animal Models	Hepadnavirus	Viral Entry	Infection/Replication	cccDNA Formation	Immune Status	Inbred/Outbred	Gene Modification	References
Chimpanzee	HBV	Yes	infection	Yes	immunocompetent	No	-	[51,52,53,54,55,56,57]
Tupaia	HBV	Yes	infection	Yes	immunocompetent	No	-	[34,40,58]
Woodchuck	WHBV	Yes	infection	Yes	immunocompetent	No	-	[48,59,60]
Duck	DHBV	Yes	infection	Yes	immunocompetent	No	-	[49,61,62]
Woolly monkey	WMHBV	Yes	infection	Yes	immunocompetent	No	-	[50,63,64]
HBV transgenic mouse model	HBV	No	-	No	Immunocompetent, tolerance to HBsAg	Yes	PreS1, S and x transgene	[65,66,67]
	HBV	No	-	No	immunocompetent	Yes	x transgene	[68,69]
	HBV	No	-	No	Immunocompetent, tolerance to HBeAg, HBcAg	Yes	PreC/C transgene	[70]
	HBV	No	replication	No	Immunocompetent, tolerance to HBV	Yes	1.1 mer genome transgene	[71]
	HBV	No	replication	No	Immunocompetent, tolerance to HBV	Yes	1.2 mer genome transgene	[71]
	HBV	No	replication	No	Immunocompetent, tolerance to HBV	Yes	1.3 mer genome transgene	[71,72,73]
	HBV	No	replication	Yes	Immunocompetent, tolerance to HBV	Yes	HNF1 α^−/−^/1.3×HBV-C57BL/6/Sv/129	[46]
	HBV	Yes	replication	No	Immunodeficient	Yes	hNTCP/BAC/1.3×HBV-NRG	[44]
Human liver chimeric mouse model	HBV	Yes	infection	Yes	Immunodeficient	Yes	Alb-uPA/*Rag*2+hHep	[74]
	HBV	Yes	infection	Yes	Immunodeficient	Yes	cDNA-uPA/SCID+hHep	[75]
		Yes	infection	Yes	Immunodeficient	Yes	*Fah^−/−^ /Rag2^−/−^ / IL2rγ^−/−^* (FRG)+hHep	[76,77,78]
	HBV	Yes	infection	Yes	Immunodeficient	Yes	*Fah^−/−^NODRag1^−/−^ IL2rγc^null^* (FNRG)+hHep	[79,80]
	HBV	Yes	infection	Yes	Immunodeficient	Yes	HSV*tk*-*NOG*(TK-NOG)+hHep	[81,82]
Dual chimeric mouse model	HBV	Yes	infection	Yes	HIS	Yes	*Fah^−/−^ /Rag2^−/−^ / IL2rγ^−/−^* (FRG)+HSC+hHep	[83]
	HBV	Yes	infection	Yes	HIS	Yes	*Fah^−/−^ /NODRag1^−/−^ / IL2rγ^−/−^* (FNRG) +HSC+hHep	[83]
	HBV	Yes	infection	Yes	HIS	Yes	*BALB/c Rag2*^−/−^*Il2rγ*^−/−^*Sirpa*^NOD^Alb-uPA^tg/tg^ (BRGS-uPA)+HSC+hHep	[84]
	HBV	Yes	infection	Yes	HIS	Yes	*HLA-A2 NOD-SCID-IL2rγ^−/−^*(A2/NSG)+HSC+hHep	[85]
	HBV	Yes	infection	Yes	HIS	Yes	*Fah^−/−^Rag2^−/−^IL-2Rγc^−/−^ SCID* +hBMSC+hHep	[86]

HIS, human immune system; SCID, severe combined immunodeficient; HSC, hematopoietic stem cell; hHep, human hepatocytes; hBMSC, human bone marrow mesenchymal stem cell.

**Table 3:** HBV genome expression systems in mice. Four major HBV genome expression systems utilized in mice for HBV research, along with their advantages and limitations.

Model	Features	Advantages	Disadvantages	References
1.3×HBV tg mice	Contains 1.3×HBV integrated into murine genome	HBV genome is integrated in the mice Produces infectious HBV, including HBV DNA, HBcAg, and HBsAg	Mice are immunologically tolerant to HBV Does not result in liver injury Does not form cccDNA	[71,112]
HDI-based replication-competent HBV tg mice	HBV replicons, i.e., 1.2×, 1.3×HBV or HBVcircle genomes, are hydrodynamically injected into mice through tail vein injection	Produces infectious HBV, including HBV DNA, HBcAg, and HBsAg Can allow cccDNA-like formation (i.e., HBV circle) Can be administered to immunocompetent mice	Sustained in cells for up to 6 months post inoculation Expressed in 10–25% of murine hepatocytes Can establish liver fibrosis with appropriate vector HBV genotype alters viral persistence	[128,129,130]
Adeno-HBV tg mice	Adenovirus vectors containing HBV genome are injected into mice	Produces infectious HBV, including HBV DNA, HBcAg, and HBsAg Expressed in ~90% of murine hepatocytes	Mice become immunologically tolerant to HBV due to altered T cell profile (advantage for immunotolerant studies) Can establish liver fibrosis No detectable cccDNA formation	[126,127,135]
AAV-HBV tg mice	AAV vectors containing HBV genome are injected into mice	Produces infectious HBV, including HBV DNA, HBcAg, and HBsAg Sustained in cells for at least one year Can be administered to immunocompetent mice	Expressed in ~60% of murine hepatocytes Mice become immunologically tolerant to HBV due to altered T cell profile (advantage for immunotolerant studies) Can establish liver fibrosis No detectable cccDNA formation or form cccDNA conceivably not through canonical rcDNA repair steps	[122,124,125]

Tg, transgenic; Adeno, adenovirus; AAV, adeno-associated virus; HBcAg, HBV core antigen; HBsAg, HBV surface antigen; cccDNA, covalently closed circular DNA.

## Data Availability

Not applicable.
